# The Significance of the Dysregulation of Canonical Wnt Signaling in Head and Neck Squamous Cell Carcinomas

**DOI:** 10.3390/cells9030723

**Published:** 2020-03-15

**Authors:** Jarosław Paluszczak

**Affiliations:** Department of Pharmaceutical Biochemistry, Poznan University of Medical Sciences, ul. Swiecickiego 4, 60-781 Poznan, Poland; paluszcz@ump.edu.pl; Tel.: +48-61-854-6624

**Keywords:** head and neck cancer, Wnt signaling, β-catenin

## Abstract

The knowledge about the molecular alterations which are found in head and neck squamous cell carcinomas (HNSCC) has much increased in recent years. However, we are still awaiting the translation of this knowledge to new diagnostic and therapeutic options. Among the many molecular changes that are detected in head and neck cancer, the abnormalities in several signaling pathways, which regulate cell proliferation, cell death and stemness, seem to be especially promising with regard to the development of targeted therapies. Canonical Wnt signaling is a pathway engaged in the formation of head and neck tissues, however it is not active in adult somatic mucosal cells. The aim of this review paper is to bring together significant data related to the current knowledge on the mechanisms and functional significance of the dysregulation of the Wnt/β-catenin pathway in head and neck tumors. Research evidence related to the role of Wnt signaling activation in the stimulation of cell proliferation, migration and inhibition of apoptosis in HNSCC is presented. Moreover, its role in promoting stemness traits in head and neck cancer stem-like cells is described. Evidence corroborating the hypothesis that the Wnt signaling pathway is a very promising target of novel therapeutic interventions in HNSCC is also discussed.

## 1. Introduction

Head and neck squamous cell carcinoma (HNSCC), which is most frequently located in the oral cavity, lip, pharynx or larynx, is one of the most common types of cancer, comprising around 4% of new cancer cases each year. HNSCC incidence is higher in men than in women [1,2]. The development of HNSCC is strongly associated with long-term tobacco use, excessive consumption of strong alcohols or, especially in the case of oropharyngeal tumors, the infection with human papilloma virus (HPV), usually HPV type 16 or 18 [3]. Despite relatively easy access for clinical inspection, these tumors are frequently detected at a late stage, when therapeutic options are less effective in curing patients, who are then at a greater risk of the development of recurrent tumors or metastasis. Thus, the overall survival rates in this group of patients remain relatively low (~50%), especially when patients are diagnosed with advanced stages of the disease [4,5]. There is a need for novel biomarkers which could improve the clinical management of HNSCC patients, including better prognostication and disease monitoring. Moreover, the development of new therapeutic options is also necessary for the improvement of treatment outcomes. Standard surgery, radiotherapy and/or chemotherapy show limited efficacy in patients with advanced stages of the disease. The introduction of molecular targeted therapy may improve clinical response. However, the use of the antagonist of EGFR (cetuximab) showed only moderate effectiveness, which was related, in part, to the development of drug resistance [6]. The recently introduced immunotherapy using pembrolizumab, which activates T lymphocytes by blocking the interaction between PD-1 receptor and PD-L1, may be beneficial in patients with advanced, metastatic or recurrent HNSCC [7]. However, other therapeutic options are also necessary for the better clinical management of HNSCC patients. To that end, molecular alterations observed in HNSCC need to be studied in detail, in order to point to promising drug targets.

## 2. Mechanisms of Wnt/β-Catenin Pathway Activation in HNSCC

Recent genomic analyses have shown the prevalence of genetic driver alterations in HNSCC, which most frequently affect genes associated with receptor tyrosine kinase (e.g., EGFR, MET, KIT), PI3K, p53, Notch or Hippo signaling pathways. On the other hand, genetic alterations in genes related to canonical Wnt signaling were rarely detected in HNSCC [8,9]. In contrast to colorectal cancers, which show molecular alterations driving Wnt signaling activation in around 90% of cases [9], HNSCC were shown to lack *CTNNB1* mutations and *APC* mutations were present infrequently [10,11,12,13,14,15,16]. The mutations of *FAT1* tumor suppressor, which encodes a protocadherin protein that binds and inactivates β-catenin, were detected in some cases of HNSCC [17]. However, the activation of the Wnt/β-catenin pathway in HNSCC seems to be more prevalent than it is suggested by genetic findings, due to cross-talk with other molecular alterations, which can lead to pathway cross-activation. Indeed, it has been shown that β-catenin can be activated via enhanced EGFR or PI3K signaling, which belong to the most frequently dysregulated signaling pathways in HNSCC. In this regard, elevated EGFR expression was associated with delocalized β-catenin expression [13]. In another study, the nuclear translocation of β-catenin correlated with high expression of EGFR in oral squamous cell carcinoma (OSCC) samples [18]. The stabilization of membrane-bound EGFR by preventing its endocytosis may lie behind galectin-mediated stimulation of Wnt/β-catenin pathway activity [19]. Other studies have shown that galectin-3-mediated induction of the Wnt pathway resulted from Akt-dependent phosphorylation and inactivation of GSK-3β [20]. The treatment of OSCC cells with epidermal growth factor increased the level of phosphorylation of β-catenin at tyrosine residues, leading to its dissociation from E-cadherin and nuclear translocation. It also stimulated β-catenin-dependent reporter gene expression [18]. Additionally, in a study on the immortalization of primary oral keratinocytes, the introduction of a mutated version of *TP53* (p53R(175)H missesnse mutation) led to a significant induction of a gene expression profile matching Wnt/β-catenin pathway activation [21].

**Figure 1:**
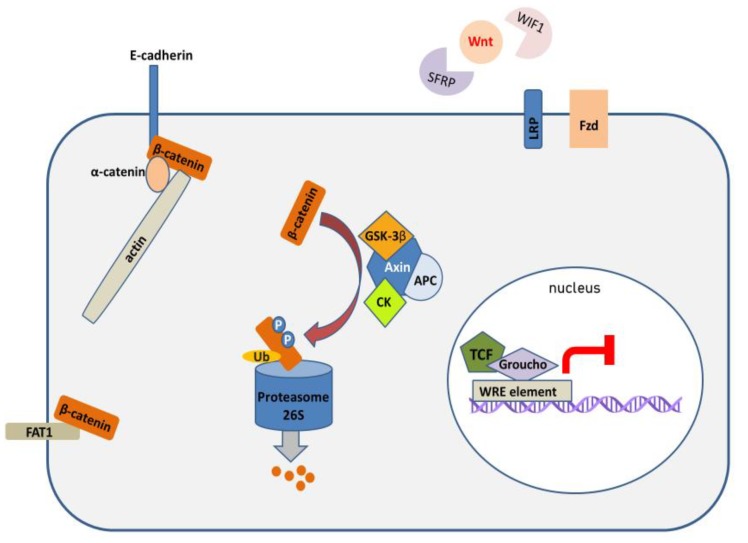
The fates of β-catenin, when the canonical Wnt pathway is not activated. Extracellular Wnt ligands are sequestered by antagonistic proteins (SFRPs, WIF1) and cannot bind to FZD/LRP receptors. Membrane-bound β-catenin takes part in cell-cell adhesion, together with E-cadherin and α-catenin. Cytoplasmic β-catenin is phosphorylated by the components of the destruction complex (CK, GSK-3β) and targeted for ubiquitin-mediated proteasomal degradation, and thus cannot translocate to nucleus and activate transcription. TCF/LEF transcription factors form complexes with suppressors of β-catenin-dependent transcription (e.g., Groucho) and cannot stimulate the expression of WRE-regulated genes. WRE—Wnt response element, CK—casein kinase 1.

**Figure 2:**
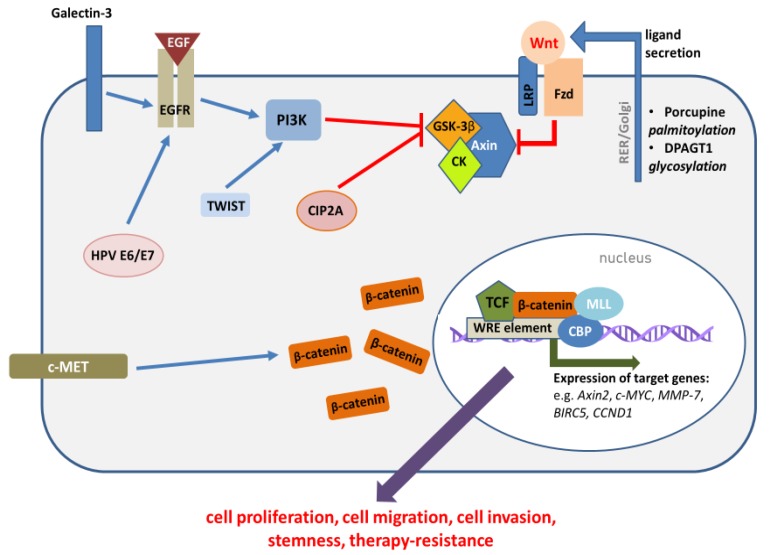
The activation of the canonical Wnt pathway in head and neck cancers is mediated by various factors. The synthesis of Wnt ligands is enhanced, which is related to the elevated activity of enzymes responsible for post-translational modifications—glycosylation and palmitoylation. The increased activity of Wnt ligands results from the reduced expression of extracellular Wnt antagonists. After secretion, Wnt ligands activate FZD/LRP receptors, which destabilizes the destruction complex, which can no longer phosphorylate β-catenin and stimulate its degradation. The destabilization of the destruction complex may also be mediated by the activation/overexpression of other molecular factors (e.g., EGFR, PI3K, c-MET, CIP2A), including HPV infection (details provided in the text). The resulting translocation of β-catenin to the nucleus activates TCF/LEF-mediated transcription of WRE-regulated genes, which relies on the cooperation with histone modifying proteins (MLL, CBP).

The central regulatory protein in the canonical Wnt pathway is β-catenin. Briefly, in unstimulated cells, cytoplasmic β-catenin undergoes proteasomal degradation, which is stimulated by its phosphorylation by the components of the destruction complex, which comprises casein kinase 1 (CK1), GSK-3β, APC and AXIN (Figure 1). On the other hand, when extracellular Wnt ligands bind to Frizzled (FZD) and LRP receptors, they lead to the inhibition of the destruction complex, and the stabilization of cytoplasmic β-catenin, which may subsequently translocate to the nucleus. In the nucleus, β-catenin binds to TCF/LEF transcription factors and induces the expression of target genes, which regulate cell survival, proliferation, cell migration and apoptosis (Figure 2) [22].

Importantly, apart from playing a key role in Wnt signaling, β-catenin has crucial functions related to the regulation of cell-cell adhesion by the formation of adherens junctions (AJ). It forms complexes with E-cadherin, and, together with α–catenin, takes part in associating E-cadherin to actin cytoskeleton in epithelial cells. Thus, although debatably, it is believed that there are two, relatively separate, pools of intracellular β-catenin—membranous and cytoplasmic, with distinct functions, which are regulated by partly independent mechanisms [23]. The canonical Wnt pathway is not active in normal epithelial cells of the head and neck region. Indeed, many immunohistochemical analyses have documented the membranous localization of β-catenin in cells of normal mucosal epithelium [24,25], while cytoplasmic or nuclear presence of β-catenin, which can mark canonical Wnt pathway activation, is infrequent in normal epithelial cells [26,27]. In contrast, dysplastic or cancerous epithelium shows a decrease in membranous β-catenin level and abnormal delocalization of β-catenin to cytoplasmic and, sometimes, also nuclear compartments [13,18,24,26,27,28,29,30,31,32,33]. A progressive loss of membranous β-catenin was reported during transition from hyperplasia to dysplasia and carcinoma [34]. On the other hand, some studies suggest that increased β-catenin nuclear localization is more frequent in dysplastic lesions than in tumors [35]. Elevated cytoplasmic expression of β-catenin in dysplastic oral and laryngeal lesions was associated with severity of dysplasia and increased cancer incidence [36,37]. Moreover, nuclear translocation of β-catenin was associated with severity of dysplasia [35] and increased expression of cyclin D1, c-MYC and MMP7 in oral dysplasia [26,38]. When it comes to cancer cases, the exact profile of β-catenin delocalization and expression level was related to tumor site and stage. Supraglottic tumors were characterized by stronger β-catenin delocalization than glottic tumors [39,40], and lip and palate tumors were not characterized by significant β-catenin dysregulation [41]. The delocalization of β-catenin was more frequently observed in poorly differentiated tumors [28,42]. In OSCC, the intracellular expression of β-catenin was associated with the lower level of expression of involucrin, a marker of keratinocyte differentiation [43]. Moreover, the delocalization of β-catenin was frequently detected in tumor invasive front [44,45]. It has been confirmed that the reduction in membranous β-catenin was associated with invasive growth, higher risk of lymph node metastasis and shorter survival [13,34,46,47]. The higher level of cytoplasmic/nuclear β-catenin delocalization detected in the surgical margin correlated with higher recurrence rate [48]. Depending on the study, the nuclear expression of β-catenin was observed in 23% of pharyngeal tumors [49], 43% of laryngeal tumors [40], 33% of tongue carcinomas [50], 78% of tongue carcinomas [51] and 17% of HPV-negative oropharyngeal carcinomas [52]. In general, the nuclear appearance of β-catenin was more frequent in poorly differentiated tumors [53]. On the other hand, some studies found only rare cases of the nuclear translocation of β-catenin in HNSCC [14,54,55,56].

The reduction in membranous β-catenin level is frequently associated with the reduction in E-cadherin level [51,55,57], which is often the consequence of the promoter methylation of the *CDH1* gene [38,58]. The loss of E-cadherin releases β-catenin from AJ complexes, which leads to the increase in cytoplasmic β-catenin level and could lead to the stimulation of β-catenin transcriptional activity. However, experiments have shown that such scenario rather takes place in cells which already show canonical Wnt activation, e.g., due to destruction complex abnormalities [23]. On the other hand, activated Wnt signaling can induce the expression of *DPAGT1*, which encodes *N*-acetylglucosamine 1-phospho-transferase [59]. This enzyme catalyzes the rate-limiting step in protein *N*-glycosylation. Glycosylated E-cadherin shows reduced ability to form stable AJ [19]. Indeed, when E-cadherin is hypoglycosylated, the growth and migration of OSCC cells is slower. Thus, the relationship is reciprocal.

The frequently observed cytoplasmic delocalization of β-catenin cannot be considered by itself as a direct evidence of canonical Wnt pathway activation in HNSCC. However, there is also other evidence corroborating this hypothesis. The loss of membranous FAT1 is observed in poorly differentiated OSCC, and this is associated with the cytoplasmic and nuclear accumulation of β-catenin, upregulation of cell growth and loss of cell adhesion [60]. Also, the elevated expression of several Wnt ligands, which activate canonical Wnt signaling, has been detected in HNSCC. The expression of WNT-7A, -10B and -13 was higher in tumor sections than in normal mucosa [61]. Also, WNT-3A, -6 and -7 were not expressed in normal oral mucosa, but their overexpression was frequently observed in OSCC [16]. While normal human oral keratinocytes lacked WNT-7B expression, its expression was observed in oral cancers, especially in less differentiated tumors. Moreover, it correlated with lymph node invasion [62]. Oral cavity tumors expressing Wnt ligands exhibited an increased level of cytoplasmic and nuclear β-catenin, and WNT-3 expression, together with nuclear β-catenin localization, were seen predominantly at the invasive tumor front [63]. High expression of WNT-3A in laryngeal tumors was associated with lymph node metastasis and shorter survival [64]. Also, dysplastic oral lesions showed much higher WNT-3A expression than normal mucosa [26,35]. Indeed, dysplastic oral keratinocytes (DOK cell line) secrete WNT-3A, what can be inhibited by the use of C59, a Porcupine inhibitor [35]. Wnt ligands are secreted as glycolipoproteins, and abnormalities in the rate of these post-translational modifications may contribute to Wnt pathway activation. In this regard, an increased expression of DPAGT1 was observed in OSCC samples, together with an increased level of WNT-3A, which is stabilized upon glycosylation. On the other hand, the siRNA-mediated silencing of *DPAGT1* was associated with a reduction in β-catenin-dependent reporter gene expression in vitro [59].

In oropharyngeal tumors, delocalized expression of β-catenin was associated with increased expression of LEF1. Importantly, LEF1 expression was absent from normal epithelium. The majority of tumors were LEF1-positive, including all advanced stage carcinomas, most recurrent cases and two thirds of tumors which were associated with death during follow-up. Interestingly, some LEF1-positive tumors were negative for β-catenin, but these tumors were positive for γ-catenin, which is redundant with β-catenin, when it comes to nuclear transcription regulation [25]. Another study found that a quarter of OSCC showed overexpression of LEF1, which was associated with poor differentiation, lymph node invasion and reduced overall survival [65]. Moreover, transcriptomic analysis of β-catenin target genes confirmed the activation of Wnt signaling in advanced and relapsed HNSCC tumors [66].

The overexpression of CIP2A, which is frequently observed in oral dysplasia and carcinoma [67,68], may also contribute to the stimulation of β-catenin transcriptional activity. CIP2A acts as an antagonist of protein phosphatase PP2A complex, which functions as a cellular regulator of β-catenin by the modulation of the destruction complex. Interestingly, depending on the cellular context and the composition of PP2A subunits, PP2A may either activate or inhibit Wnt signaling [69]. Direct evidence connecting CIP2A overexpression and β-catenin nuclear translocation is currently missing. On the other hand, the expression of SET protein, which is another PP2A inhibitor, is also elevated in HNSCC [70], which suggests that the abnormalities in this system are important players during head and neck carcinogenesis.

### 2.1. The Role of Epigenetic Mechanisms in Wnt/β-Catenin Pathway Activation

Epigenetic alterations play an important role in Wnt pathway activation in HNSCC. The activity of the pathway is regulated physiologically by a series of antagonistic proteins which downregulate Wnt signaling by sequestrating extracellular Wnt ligands (SFRP and WIF proteins), blocking the receptor complex proteins (DKKs), antagonizing intracellular signal transduction (e.g., AJUBA, DAB2) or inhibiting the formation of active β-catenin transcriptional complex (e.g., Chibby). The expression of these genes is frequently reduced by promoter methylation in cancer. Indeed, *SFRP1*, *SFRP2* and *SFRP5* genes were frequently hypermethylated in OSCC cell lines and in primary tumor samples, while their ectopic expression suppressed the growth of OSCC cells in vitro [16,71]. Additionally, *SFRP2* was found methylated in the majority of OSCC samples, in contrast to corresponding normal mucosa, and its overexpression reduced tumor growth in vivo [72]. It was shown that the reduction in *SFRP1* and *SFRP5* expression, which was induced by gene promoter methylation, was associated with cytoplasmic/nuclear delocalization of β-catenin in oral lesions [73]. Apart from *SFRP1* and *SFRP2*, also *WIF1*, *DKK1* and *PPP2R2B* were methylated in LSCC cell lines and primary tumors [74]. The expression of Wnt ligand antagonist WIF1 is observed in normal oral epithelium, suggesting that WIF1 plays a role in the inhibition of canonical Wnt signaling in healthy mucosa. However, the expression of WIF1 was much lower in OSCC samples [59], and this was related to the methylation of *WIF1* promoter [73]. The methylation of *WIF1* in OSCC was found to be cancer-specific when compared to normal mucosa [73,75]. The aberrant methylation of *SFRP2* and *WIF1* was shown to be already present in dysplastic lesions and the rate of methylation increased with progression to OSCC [76]. Another study showed that the methylation of *WIF1* predicted shorter survival in OSCC patients [77], however such a correlation was not found in tongue carcinomas [78]. Also, the expression of Chibby was decreased in LSCC samples, when compared with normal laryngeal mucosa, and this was mediated by abnormal *CBY* promoter methylation [79].

Recent studies have shown the important involvement of regulatory RNA molecules in the regulation of Wnt/β-catenin signaling, and abnormalities in the expression of miRNA were implicated in the activation of the Wnt pathway. The epigenetic silencing of miR-329 and miR-410, which may be induced by arecoline, the carcinogenic ingredient of betel quid, was associated with the upregulation of WNT-7B expression and the induction of expression of *CCND1* and *c-MYC*. It was found that arecoline leads to epigenetic silencing of these miRNAs, and the induction of their re-expression by a combined treatment with DNA methyltransferase and histone deacetylase inhibitors reduced cell proliferation and migration [62]. Also, miR-29a acts as a suppressor of canonical Wnt signaling, and it is frequently down-regulated in OSCC [80]. In contrast, miR-21 promoted the Wnt/β-catenin pathway by reducing the expression of *DKK2*, which acts as the antagonist of LRP6 co-receptor. Oral carcinomas were characterized by increased expression of miR-21 and loss of *DKK2* expression [81]. Moreover, several long non-coding RNA molecules were also implicated in the regulation of Wnt signaling. The upregulation of *MINCR*, *PLAC2* and *UCA1* was frequently observed in head and neck cancers, and functionally, it promoted malignant progression by the activation of Wnt/β-catenin pathway [82,83,84]. The level of *MINCR* expression correlated with lymph node metastasis and shorter survival of OSCC patients. The knockdown of *MINCR* reduced β-catenin-dependent reporter gene expression, and also reduced the expression of Wnt target genes—*CCND1* and *c-MYC*—but elevated the level of E-cadherin. This resulted in reduced cell viability, reduced cell migration and invasion, and cell cycle arrest and increased apoptosis in cancer cell lines [82]. The elevated expression of *PLAC2* in tongue carcinoma cells resulted from the enrichment of its promoter region in H3K27 acetylation, which was dependent on CBP acetyltransferase activity. *PLAC2* knockdown also diminished cell proliferation and migration [83]. The overexpression of *UCA1* in laryngeal cancer cells was associated with reduced GSK-3β phosphorylation and elevated β-catenin level. The inhibition of Wnt signaling using IWP-2, a Porcupine inhibitor, reduced the stimulatory effects of *UCA1* overexpression on cell proliferation and migration [84].

### 2.2. The Role of HNSCC Etiological Factors in Wnt/β-Catenin Pathway Activation

HNSCC is largely induced by environmental carcinogens, and it has been shown that both HPV infection and chemical carcinogens can stimulate canonical Wnt signaling in epithelial cells. Based on the analysis of the level of expression of β-catenin target genes, it has been suggested that HPV-negative tumors show stronger activation of Wnt/β-catenin signaling, at least in patients using tobacco [85]. Also, the overexpression of ROR2, which is associated with the activation of the non-canonical Wnt pathway, was observed in HPV-positive tumors [86]. On the other hand, nuclear translocation of β-catenin was frequently observed in HPV-positive tonsillar cancers, in contrast to HPV-negative cancers, in which nuclear localization of β-catenin was more prevalent in metastases than in primary tumors [87]. Also, HPV status correlated with the loss of membranous β-catenin and the presence of nuclear β-catenin in oropharyngeal tumors [47,88]. It has been shown that human papillomavirus E6 and E7 oncogenes are involved in the stimulation of translocation of β-catenin to cell nucleus and the induction of β-catenin/TCF-dependent transcription of target genes, which could be reversed by E6 and E7 silencing. Mechanistically, this stimulating effect was associated with the inhibition of Siah-1-dependent β-catenin proteasomal degradation [89]. Other studies provided evidence, that HPV infection leads to β-catenin stabilization via the activation of receptor tyrosine kinase signaling. It was shown that HPV16 infection led to the phosphorylation of EGFR at Tyr 1173, which was responsible for the induction of β-catenin nuclear translocation and could be inhibited by the use of the EGFR inhibitor—erlotinib [47]. On the other hand, the sole overexpression of E6/E7 could not lead to the activation of the Wnt/β-catenin pathway. However, the co-expression of E6/E7 together with ErbB-2 induced the neoplastic transformation of normal oral epithelial cells, which was associated with the downregulation of E-cadherin and the nuclear translocation of catenins [90].

4-Nitroquinoline-1-oxide (4-NQO) is a synthetic compound mimicking the action of tobacco carcinogens, and is used for the experimental induction of tongue carcinomas in rats. It leads to the progressive development of oral lesions, from hyperplasia through dysplasia to carcinoma, which show similar characteristics to human oral cancers [91]. The dysplastic lesions which developed after 4-NQO administration were characterized by cytoplasmic delocalization and nuclear translocation of β-catenin [92]. The cytoplasmic/nuclear expression of β-catenin in dysplastic and cancer cells was shown to be associated with its phosphorylation at tyrosine residues, which led to the release of β-catenin from complexes with E-cadherin [93]. It is widely recognized that the concurrent exposure to ethanol greatly potentiates the carcinogenic effects of tobacco chemicals [94]. It has been observed that the administration of 4-NQO increased the level of β-catenin in tongue epithelium, while the combined treatment with 4-NQO and ethanol additionally significantly decreased the inhibitory phosphorylation of β-catenin [95]. This suggests stronger activation of β-catenin-dependent transcription; however, this was not verified by the authors. Additionally, in a hamster model of buccal pouch carcinomas, the administration of DMBA (7,12-dimethylbenz[a]anthracene) led to a progressive increase in the level of Wnt-1, Wnt-3 and Wnt-4 ligands, and the accumulation of nuclear β-catenin. Moreover, a progressive decrease in the expression of Wif1 antagonist was observed, what could contribute to Wnt/β-catenin pathway stimulation. Importantly, the expression of β-catenin target genes—*Ccnd1*, *Mmp-2* and *Mmp-9*, was also elevated [96]. Finally, the neoplastic transformation of lung epithelial cells induced by cigarette smoke extract was associated with nuclear accumulation of β-catenin and a strong stimulation of β-catenin-dependent reporter gene expression. Moreover, the stimulation of Wnt/β-catenin was, at least in part, dependent on the activation of Hedgehog signaling, because the transcriptional activity of β-catenin could be significantly reduced following treatment with Hedgehog pathway inhibitor cyclopamine [97].

## 3. Functional Significance of Wnt/β-Catenin Pathway Dysregulation

It is believed that, within a tumor, cancer cells can be divided into two general groups: most cells form the bulk of tumor, and these cells are usually targeted by classical chemotherapy, while the group of tumor propagating cells, or the so called cancer stem-like cells (CSC), remain resistant to standard chemotherapeutics. Thus, it is currently believed that targeting CSC is the key to successful chemotherapy. Recent findings have linked abnormalities in canonical Wnt signaling to HNSCC tumor propagating cells. Indeed, higher activity of the canonical Wnt pathway correlated with stem cell traits, e.g., enhanced expression of CD44 and LGR5 [85]. LGR5 is the receptor for proteins of the R-spondin family, which synergize with Wnt ligands in the stimulation of the Wnt/β-catenin pathway and play a role in adult stem cell regulation. The expression of LGR5 was increased in oral dysplasia and oral carcinoma [98]. On the other hand, the phenotype of cancer stem-like cells derived from HNSCC patients was associated with c-MET receptor-dependent activation of canonical Wnt signaling. c-MET was identified as a marker of self-renewal, and it induced the expression of FZD8 receptor via the activation of ERK/AP-1 signaling. The pharmacologic inhibition of c-MET eliminated cancer stem cells, and this was related to the reduction in FZD8 level and the downregulation of Wnt signaling. The c-MET inhibitor reduced the level of nuclear β-catenin and the level of β-catenin-dependent gene expression [99]. In another study, side population cells derived from HNSCC patients, which were enriched in cancer stem cells and were characterized by high invasiveness and enhanced tumorigenicity in vivo, also showed the abnormal activation of canonical Wnt signaling [100]. Laryngeal carcinoma CSC expressing CD133 were also characterized by Wnt pathway activation [101]. HNSCC cancer stem cells are frequently defined as cells expressing CD44 and ALDH1. Such population of cells was found to express nuclear β-catenin, which was associated with their self-renewal capacity, while the knockdown of β-catenin reduced stemness and tumorigenicity [102]. It has been found that Wnt activation was greatest at the boundary between epithelial and stromal compartments, and both cancer cells and cancer-associated fibroblasts mediated reciprocal stimulation by paracrine signals, mainly WNT-3A. In this model, the activation of canonical Wnt signaling induced stemness and increased invasive potential [66]. Several other studies also documented the relationship between Wnt signaling and cell invasion. WNT-1-mediated β-catenin activation was associated with reduced cell apoptosis and enhanced cell invasion in HNSCC cells [103]. The siRNA-mediated downregulation of *WNT-1* expression in oral cancer cells inhibited Wnt/β-catenin signaling and reduced the expression of Vimentin, a marker of epithelial-to-mesenchymal transition [104]. The introduction of mutated β-catenin into oral cancer cells induced the expression of Wnt target genes, including the upregulation of *MMP-7*, which resulted in enhanced cell migration and invasion [105]. Additionally, TRAF4-mediated activation of the Wnt/β-catenin pathway in oral cancer cells led to increased expression of c-MYC, cyclin D1, MMP-2 and MMP-9, which was associated with enhanced cell growth and also cell migration and invasion, but it was reversed by siRNA-mediated silencing of β-catenin [106]. All this evidence suggests that targeting the Wnt/β-catenin pathway may be utilized to significantly diminish the number of tumor propagating and invading cells, in order to prevent tumor spread or recurrence.

Many studies have analyzed the significance of therapeutic targeting of the Wnt/β-catenin pathway in HNSCC. Due to the central role of β-catenin in mediating Wnt signal transduction and transcription activation, this protein attracted much attention when it comes to assessing the anti-cancer effects of its downregulation. The induction of β-catenin transcriptional activity in HNSCC cell lines using lithium chloride, an inhibitor of GSK-3β, led to an increased cell proliferation rate, enhanced cell migration and invasion and a reduced rate of apoptosis [80]. Accordingly, the siRNA-mediated silencing of β-catenin in tongue cancer cells led to G0/G1 arrest, growth inhibition, decreased cell migration and increased apoptosis [51,107]. Moreover, the knockdown of *CTNNB1* significantly reduced tumor growth in xenograft mice [107] and led to the induction of both apoptosis and autophagy in laryngeal cancer cells [108]. Because HNSCC cancer cells produce and secrete Wnt ligands, in contrast to normal mucosal cells, the inhibition of Wnt signaling can be affected by ligand sequestration. Indeed, antibodies against WNT-1 or WNT-10B reduced HNSCC cell viability and β-catenin-dependent reporter gene expression. Moreover, growth inhibition could be exerted with the use of recombinant SFRP1 protein [109].

Arecoline-induced β-catenin overexpression in oral epithelial cells was reduced by ERK and PI3K small molecule inhibitors [33]. It has been observed that the overexpression of TWIST1 transcription factor increased β-catenin-dependent transcription of *c-MYC* and *MMP-2*, which resulted in increased cell invasion. TWIST1-induced Wnt activation was mediated by PI3K, which phosphorylated and inactivated GSK-3β, leading to β-catenin stabilization. Thus, TWIST1-dependent activation of β-catenin was inhibited by a PI3K inhibitor [110]. Emodin is a natural anthraquinone, which reduced TWIST1-induced epithelial-to-mesenchymal transition and invasiveness by the inhibition of the PI3K/Akt/β-catenin pathway in hypopharyngeal cancer cells [111]. These findings underline the importance of targeting the cross-talk between PI3K/Akt and Wnt signaling in HNSCC.

The silencing of galectin-3 in tongue cancer cells led to the induction of β-catenin degradation, which resulted in decreased level of MMP-9 expression and diminished cell migration and invasion capacity [20]. Interestingly, galectin-3-mediated cell proliferation, migration and invasion was reduced by treatment with DKK1, which suggested that the upregulation of WNT-1 expression by galectin-3 may play an important role in Wnt/β-catenin pathway activation in galectin-3-positive cancer cells [112].

The activation of canonical Wnt signaling in HNSCC has been shown to result from the overexpression of several other proteins as well. CUL4B, which takes part in protein ubiquitination, may activate the Wnt/β-catenin pathway by reducing the level of DKK1 and PPP2R2B antagonists. CUL4B was overexpressed in around 50% of HNSCC cases. It has been shown that the knockdown of CUL4B reduced the level of active β-catenin, decreased β-catenin-dependent reporter gene transcription and led to the reduction in the level of expression of cyclicn D1, c-MYC, MMP-7 and vimentin, while it increased the level of expression of E-cadherin. It resulted in reduced cell growth and diminished cell migration and invasion. Importantly, the effects exerted by CUL4B overexpression were abolished by treatment with a tankyrase inhibitor—XAV-939, which blocks the canonical Wnt pathway by preventing AXIN degradation and subsequent destabilization of β-catenin destruction complex, which are stimulated by active tankyrase [113]. Also, glutamate decarboxylase 1 (GAD1), the key enzyme in GABA synthesis, was found to be frequently overexpressed in OSCC. It has been observed that the knockdown of *GAD1* suppressed the nuclear translocation of β-catenin and reduced the level of MMP-7, which led to reduced cell migration and invasion [114]. On the other hand, the loss of expression of psoriasin, which was detected in poorly differentiated, late-stage oral cavity tumors, was associated with β-catenin activation. Psoriasin is normally engaged in the induction of GSK-3β-independent and Siah-1-mediated degradation of β-catenin. When expressed, it reduces cell proliferation, migration and tumor growth. The downregulation of psoriasin was associated with the acquiring of cell invasiveness and tumor progression [115].

Several natural agents have been shown to exert anti-cancer effects by attenuating Wnt signaling in HNSCC. Ellagic acid prevented the formation of DMBA-induced tumors in the buccal pouch hamster model by downregulating the expression of Frizzled and Dishevelled-2 and reducing the activation of the Wnt/β-catenin pathway [116]. Astaxanthin reduced the level of nuclear β-catenin through the inhibition of ERK/Akt kinases and reduced phosphorylation of GSK-3β. Similar to ellagic acid, astaxanthin exerted chemo-preventive effects in the hamster buccal pouch cancer model [117]. Honokiol reduced stemness in oral cancer cells. It decreased the number of stem-like side population cells by affecting CD44 and Wnt pathway signaling. It inhibited the transcriptional activity of β-catenin and diminished the level of expression of survivin, cyclin-D1 and c-MYC, and the level of β-catenin-related epithelial-to-mesenchymal markers [118].

The inhibition of canonical Wnt signaling was also exerted by several pleiotropic therapeutics. Pyrithione zinc inhibited PI3K/Akt signaling and the downstream activation of the Wnt/β-catenin pathway. It reduced the level of expression of c-MYC and cyclin D1. It reduced cell proliferation, migration and invasion and led to the induction of apoptosis and reduced xenograft tumor growth [119]. Nano-formulated quinacrine induced apoptosis in oral cancer stem-like cells by the joint inhibition of Wnt/β-catenin and Hedgehog/Gli1 pathways [120]. All-trans retinoic acid is a powerful agent stimulating cell differentiation, which exerts chemo-preventive effects in head and neck carcinogenesis. Also, this compound inhibited the proliferation of head and neck cancer stem-like cells by the inhibition of β-catenin activity [121]. The antihelminthic drug niclosamide has also been shown to inhibit the Wnt pathway in head and neck cancer cells [122,123]. It led to a reduction of the level of Dishevelled-2 and β-catenin. Also, it reduced colony formation capacity and decreased the level of expression of stemness markers in oral cancer cells [123].

Several potential molecular targets have been proposed for the effective attenuation of the Wnt/β-catenin pathway in head and neck cancer cells [124]. Since HNSCC cells have been shown to secrete Wnt ligands, one of the strategies is to prevent the formation of mature Wnt proteins by blocking their acylation. Porcupine is a membrane-bound acyltransferase, which attaches palmitic acid residues to Wnt proteins. It has been shown that targeting Porcupine is an effective strategy of inhibition of canonical Wnt signaling in HNSCC. Small molecule inhibitors of Porcupine activity have been recently developed. LGK974 inhibited β-catenin-dependent gene expression and reduced xenograft tumor growth in mice [125]. Another Porcupine inhibitor, IWP-2, also reduced β-catenin-dependent gene expression, inhibited migration and induced apoptosis in HNSCC cells [124]. The inhibition of Porcupine by C59 blocked the secretion of WNT-3A by dysplastic oral keratinocytes and reduced nuclear β-catenin level, leading to the inhibition of target gene (*CCND1*, *BIRC5*) expression [35]. Moreover, Porcupine inhibitors (LGK974 and C59) impaired the initiation and growth of HPV-driven cutaneous SCC and reduced the expression of CSC markers [126].

RAP1 is another protein which enhances the nuclear translocation of β-catenin and facilitates its transcriptional activity. The silencing of *RAP1* diminished the level of β-catenin activation by lithium chloride or WNT-3A. It decreased MMP-7 expression and blocked cell invasion in HNSCC cell lines [127]. This suggests that it also might serve as a molecular target for Wnt pathway inhibition. Different components of the β-catenin transcriptional complex constitute good molecular targets for Wnt inhibition. Small molecules (iCRT-3 and PKF118-310) which inhibit the interaction between β-catenin and TCF transcription factors, resulted in diminished Wnt signaling in HNSCC cells [128,129]. The activation of β-catenin-dependent transcription requires the recruitment of epigenetic writers, which modify chromatin. The CBP histone acetyltransferase is crucial in stimulating the expression of Wnt target genes. CBP inhibition reduced the expression of Wnt target genes, decreased cell migration and induced cell cycle arrest and apoptosis in HNSCC cells [124]. Moreover, its inhibition with ICG-001 led to significant anti-cancer effects—it reduced the growth of oral tumors and inhibited metastasis in vivo. Moreover, detailed analyses have shown that this compound selectively targeted cancer stem-like cell subpopulations [130,131]. Canonical Wnt signaling was shown to be necessary for the induction of pluripotency by the creation of permissive chromatin in stemness gene promoters. This was dependent on the formation of a complex between TCF4, β-catenin, CBP and MLL1. MLL1 is a histone methyltransferase which catalyzes H3K4 methylation. Together, these proteins were shown to interact in promoting self-renewal in HNSCC tumor propagating cells, which were highly proliferative and highly tumorigenic. The formation of this complex could be blocked by ICG-001 [130] or LF3, an inhibitor of interaction between β-catenin and TCF4 [132], which resulted in the loss of the capacity of tumor propagating cells for self-renewal and reduced tumor formation in vivo.

HNSCC tumors are frequently resistant to standard chemotherapy, and the mechanisms of chemoresistance are poorly understood. Different observations have shown that the activation of the Wnt pathway may be responsible for chemo- and radio-resistance in HNSCC, which may be contradicted with the use of Wnt pathway inhibitors. The knockdown of β-catenin sensitized HNSCC stem-like cells to cisplatin [102]. 14-3-3σ stabilizes GSK-3β and reduces TCF/LEF transcriptional activity. The downregulation of 14-3-3σ by gene promoter methylation was responsible for the induction of cisplatin resistance in tongue cancer cells. This was mediated by the activation of the Wnt pathway [133]. In the same manner, the induction of Wnt signaling by the inhibition of GSK-3β using lithium chloride led to enhanced DNA repair, evasion of apoptosis and cisplatin resistance [48]. The acquired cisplatin resistance in tongue cancer cells was associated with the induction of WNT-2B and GLUT1 overexpression, and the knockdown of WNT-2B sensitized resistant cells to cisplatin treatment and reduced colony formation in vitro and tumor growth in vivo [134]. Moreover, Wnt signaling is responsible for the stimulation of DNA repair upon radiation-induced damage. In this regard, the silencing of β-catenin increased radio-sensitivity of HNSCC cells by preventing the induction of Ku70/Ku80 DNA repair proteins [135]. In line with this, the stimulation of β-catenin nuclear translocation led to elevated expression of Ku70 and Ku80 proteins and the induction of radio-resistance via a cyclooxygenase-2-dependent mechanism [136]. Thus, targeting Wnt signaling may be used for the sensitization of HNSCC towards chemotherapy and radiotherapy. On the other hand, the important side effects associated with radiotherapy result from the damage of irradiated normal epithelium. Interestingly, it has been shown that the activation of canonical Wnt signaling by R-spondin-1 could contribute to the protection of normal cells from detrimental radiation-induced damage and prevent the development of oral mucositis [137]. All of the above indicate that abnormalities in Wnt/β-catenin signaling consist not only of potential diagnostic markers, but, most of all, constitute promising therapeutic targets.

## 4. Conclusions

Much evidence has accumulated pointing to the significance of activated Wnt/β-catenin signaling in driving carcinogenesis in the head and neck region. The most important molecular alterations related to Wnt pathway activation in HNSCC and promising therapeutic targets are listed in Table 1. Based on its proven connection with the induction of stemness traits, it seems that targeting the Wnt pathway may be effective in reducing cancer burden in HNSCC patients. However, several questions remain unanswered. The real extent of the activation of Wnt signaling in patients is not well characterized, thus it is not clear how to identify patients who would benefit from such molecular targeted therapy. The activation of the Wnt/β-catenin pathway depends on different triggers. This would be advantageous if a model, in which these molecular triggers converge in Wnt activation, would be accurate. However, this is rather more complicated, because of the many interconnections between dysregulated molecular pathways. Thus, it would be important to analyze the head and neck cancer signalome, in order to better understand the molecular networks that drive HNSCC and how they operate in different types of cancer cells. This would allow to better define the molecular factors whose analysis could be used for the choice of drugs for precision medicine. In this context, the clinical utility of molecular therapeutics targeting Wnt signaling, especially in combination with other anti-cancer agents, needs to be precisely assessed.

## Figures and Tables

**Table 1:** Key molecular alterations related to canonical Wnt pathway activation in HNSCC.

Gene/Protein	Type of Alteration	Molecular Effects	Functional Significance	Ref.
Drivers of Wnt activation				
*WNT* ligands	overexpression	translocation of β-catenin	lymph node invasion	[61,62,63,64]
*APC*	mutational loss	stabilization of β-catenin	enhanced cell growth	[10,11,12,13,14,15,16]
*FAT1*	mutational loss	reduced sequestration of β-catenin	enhanced cell growth, loss of cell adhesion	[60]
*CDH1*	(epi)mutational loss	release of β-catenin from cell-cell junctions	enhanced cell growth, loss of cell adhesion	[51,55,57,58]
*EGFR*	overexpression	stabilization and nuclear translocation of β-catenin	enhanced cell proliferation	[13,18]
*c-MET*	overexpression	Wnt activation via FZD8	increased stemness	[99]
*SFRP1-5*	epigenetic silencing	reduced Wnt ligand sequestration	worse prognosis	[73]
*WIF-1*	epigenetic silencing	reduced Wnt ligand sequestration	worse prognosis	[73,77]
HPV *E6/E7* oncogenes	overexpression	stabilization of β-catenin	neoplastic transformation	[47,89]
β-catenin	nuclear accumulation	enhanced expression of Wnt target genes (*CCND1, c-MYC, MMP7*)	invasiveness, lymph node metastasis, recurrence, dedifferentiation	[26,28,38,42,44,45,46,47,48]
*LEF1*	overexpression	transcriptional activation	lymph node invasion	[25,65]
Targets for therapeutic inhibition of Wnt signaling				
WNT-1	knockdown	reduced Vimentin expression	inhibition of epithelial-to- mesenchymal transition	[104]
	inhibition by antibody	reduced expression of Wnt target genes	reduced HNSCC cell viability	[109]
FZD-DVL complex	niclosamide	altered gene expression	reduced stemness	[123]
β-catenin	knockdown	decreased gene expression of *CCND1, c-MYC, MMP-7*	reduced stemness	[102]
			decreased cell invasion	[106]
			cell cycle arrest, reduced cell migration, induction of apoptosis	[51,107]
			decreased cisplatin resistance	[102]
			increased radiosensitivity	[135]
Porcupine	IWP-2 inhibitor	inhibition of *UCA1*- dependent Wnt activation	reduced cell proliferation and migration	[84]
	LGK974 inhibitor	reduced Wnt target gene expression	reduced tumor growth	[125]
	C59 inhibitor	reduced secretion of WNT-3A, reduced *CCND1* and *BIRC5* expression	impaired HPV-driven transformation	[126]
CBP	ICG-001 inhibitor	altered gene expression	cell cycle arrest, induction of apoptosis, reduced stemness, tumor growth and metastasis	[130,131]
PI3K pathway	emodin	inhibition of PI3K/Akt/ β-catenin pathway	reduced cell invasiveness	[111]
	pyrithione zinc	reduced expression of *CCND1* and *c-MYC*	reduced cell proliferation and invasion, apoptosis	[119]
CUL4B	knockdown	reduced expression of *CCND1, c-MYC, MMP-7*	reduced cell growth, migration and invasion	[113]
